# Online peer support for mental health in individuals with post‐acute sequelae of COVID‐19: A pre‐post pilot study with mixed methods

**DOI:** 10.1002/pcn5.238

**Published:** 2024-08-18

**Authors:** Megumi Hazumi, Michi Miyake, Mayumi Kataoka, Kentaro Usuda, Daisuke Nishi

**Affiliations:** ^1^ Department of Public Mental Health Research, National Institute of Mental Health National Center of Neurology and Psychiatry Kodaira Tokyo Japan; ^2^ Department of Sleep‐Wake Disorder, National Institute of Mental Health National Center of Neurology and Psychiatry Kodaira Tokyo Japan; ^3^ Department of Mental Health, Graduate School of Medicine The University of Tokyo Bunkyo‐ku Tokyo Japan

**Keywords:** depression, mixed method, peer support, post‐acute sequelae of COVID‐19, pre‐post intervention

## Abstract

**Aim:**

This pilot examined the effect of online peer support on mental health problems among individuals with post‐acute sequelae of COVID‐2019 (PASC).

**Methods:**

A single‐arm pre‐post design of online peer‐support design consisting of eight sessions of 1 h per week with three to six participants and two facilitators per group was performed. Participants were recruited from online communities, social media, and medical clinics for the PASC between May and August 2023. The degrees of depression, anxiety, loneliness, social withdrawal, and self‐esteem were measured pre‐ and post‐intervention. Participants' statements during the sessions were analyzed using thematic analyses.

**Results:**

Of the 18 participants, three dropped out of the interventions, and 17 (including two participants who dropped out) completed the pre‐ and post‐intervention questionnaires. Depression severity significantly decreased in the paired *t*‐test and linear mixed model. The following interactions were extracted: conveying the same feelings, dealing with difficulties, showing empathy, enhancing the atmosphere, and adapting to suit health conditions. Impressions extracted from participating in the interventions included feelings of emotional support, a sense of bonding, changes in perspective, changes in behaviors or new actions through participation, inadequacy during sessions, and adverse effects associated with participation.

**Conclusion:**

Online peer support may be helpful in treating depression in individuals with PASC.

## Graphical abstract

This pre‐post study investigated the effects of an online peer‐support group on mental health in individuals with post‐acute sequelae of COVID‐19. Of 18 participants, 17 completed both the pre‐ and post‐intervention questionnaires, and 15 remained until the end of intervention program, which included 8 weekly 1‐h sessions. Repeated *t*‐test showed a significant decrease in depression severity from pre‐ to post‐intervention under both intention to treat and per protocol analyses.

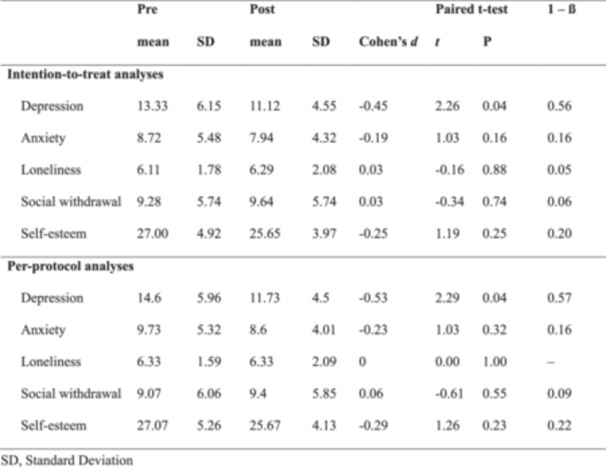

## INTRODUCTION

Post‐acute sequelae of coronavirus disease 2019 (COVID‐19) (PASC) is defined as the maintenance, relapse, or emergence of symptoms beyond 4 weeks after COVID‐19 infection, such as fatigue, brain fog, dizziness, and gastrointestinal symptoms.^1^, ^2^ Of individuals with COVID‐19, 12.44% had at least one symptom of PASC.^3^ The burden of health loss is substantial, with 35%–69% of PASC persisting for over 2 years.^4^

Mental health problems, often observed in those with PASC, are suggested to affect prolonged PASC. Approximately 23% of patients with PASC have mental health problems,^5^ and association between PASC and mental health problems has been noted.^6^, ^7^ In addition to PASC often observed in those with preexisting mental health problems,^8^, ^9^ PASC itself is suggested to contribute to mental health problems.^10^ They may be promoted by psychosocial burdens secondary induced by PASC, such as the inhabitation of work ability^11^ and stigma.^12^, ^13^ Considering mental health problems are suggested to prolong PASC from longitudinal studies,^14^, ^15^, ^16^ decreasing mental health problems among individuals with PASC is crucial.

In addition to psychological intervention,^17^, ^18^ physical rehabilitation,^18^, ^19^ exercise,^20^, ^21^, ^22^ and non‐invasive cognitive stimulation,^23^, ^24^, ^25^ online peer support is suggested to be useful for mental health problems in individuals with PASC when conducted with trained facilitators. Online peer support, recommended by several national health organizations for PASC as a strategy to address psychosocial aspects, consists of regular meetings via online video systems for individuals experiencing similar difficulties.^26^, ^27^ Considering the difficulties that individuals with PASC may face in commuting to medical institutions because of their symptoms,^28^ participating in online peer support may be a more practical option than face‐to‐face peer support. According to a qualitative study, participants complemented the treatment gap by obtaining insights, behavioral engagement, diversity, social connection, and delivering or obtaining support through a peer‐support group.^29^, ^30^ The effects of peer support on mental health have also been demonstrated in other populations. Online peer support during the COVID‐19 pandemic has also been reported to be helpful for mental health in populations other than those with PASC.^31^ On particular, peer‐support groups with trained facilitators have been shown to increase the effects, as indicated by a systematic review identifying accelerators of peer‐support groups.^32^

However, to our knowledge, the effects of regular online peer‐support group meetings with trained facilitators on individuals with PASC have not yet been examined. A systematic review indicated that few studies have examined the effect of online peer support for PASC via regular meetings with trained facilitators.^33^ To date, although two studies have reported that self‐management programs containing online peer‐support sessions improve psychosocial problems,^34^, ^35^ neither examined the effect of peer‐support sessions alone. One study focused online communities for PASC providing communication services by text message or video at any given time, but how many and how much participants utilized the services were unknown.^34^ In another study, the main intervention was cognitive behavioral therapy lectures over regular sessions and an online peer‐support group that was optional and unstructured in terms of participants, times, and medium.^35^ Hence, the effects of a peer‐support group with regular sessions remain unclear. Moreover, although some experiences through peer‐support groups for PASC have been identified,^29^ it may be incomprehensive owing to the lack of information regarding negative experiences and the interactions between individuals during peer‐support sessions.

This study aimed to examine the effect of online peer support at regular meetings with trained facilitators on the mental health of individuals with PASC. Additionally, subjective experiences and impressions of online peer‐support sessions and interactions during sessions were examined using qualitative methods to explore the elements that impact the effect.

## MATERIALS AND METHODS

### Study design

This pilot study employed a pre‐ and post‐single‐arm design of online peer support for over 8 weeks. Participants responded to online questionnaires using Microsoft Forms three times: on participant agreement, before starting the intervention, and after finishing the intervention. Participants' statements during all intervention sessions were also recorded using the Zoom recording system, which was used to hold the groups and collect them for qualitative analyses.

### Participants

Recruitment was performed from May 2023 to August 2023 by posting recruitment advertisements on several social network services, such as anonymous online communities for PASC, including three LINE open chat groups containing approximately 100–900 members and a Facebook group containing approximately 30 members, X, and at a medical clinic for PASC. Regardless of the number of sessions attended and discontinuation of participation, participants who completed all three questionnaires received prepaid cards worth JPY 3000 as a reward. Inclusion criteria were (a) 18 years old or older, (b) self‐reported as having PASC, (c) a patient‐health questionnaire‐9 (PHQ‐9) score ≥5 to avoid the floor effect and more clearly assess the effectiveness of the intervention, (d) access to an online environment and devices, and (e) having an appropriate talking environment, such as a private room. Exclusion criteria were (a) difficulty in attending all sessions at the time of obtaining consent and (b) the score of the item regarding suicidal intention at PHQ‐9 ≥ 2. Participants who were absent from more than half the sessions were identified as dropouts.

The sample size was required to be 18 or more, based on the sample size criteria for pilot studies.^36^

### Measurements

#### Patient Health Questionnaire‐9

The Patient Health Questionnaire‐9 (PHQ‐9) was used for assessing the severity of depression, the primary outcome. It consists of nine items with a four‐level Rikert scale (0–3).^37^, ^38^ A total score of 5 or more indicates mild depression. This scale was collected at participant agreement and pre‐ and post‐intervention.

#### General Anxiety Disorder‐7

The General Anxiety Disorder‐7 (GAD‐7) scale was used to assess the severity of anxiety (a secondary outcome). It consists of seven items with a four‐level Rikert scale (0–3).^39^, ^40^ Higher scores indicate severe anxiety. Data were collected pre‐ and post‐intervention.

#### Three‐item loneliness scale

A three‐item loneliness scale was used to assess the severity of subjective loneliness (a secondary outcome), consisting of three items with a three‐level Rikert scale (1–3).^41^, ^42^ Higher scores indicate severe loneliness. Data were collected pre‐ and post‐intervention.

#### Luben Social Network Scale

The Luben Social Network Scale (LSNS) was used to assess the severity of social withdrawal (a secondary outcome). It comprises six items with a six‐level Rikert scale (0–6).^43^, ^44^, ^45^ Higher scores indicate severe social withdrawal. Data were collected pre‐ and post‐intervention.

#### The Rothenberg Self‐esteem Scale

This scale, consisting of 10 items with a four‐level Rikert scale (1–4), was used to assess the degree of self‐esteem (a secondary outcome). Higher scores indicate higher self‐esteem.^46^, ^47^ Data were collected pre‐ and post‐intervention.

#### Others

Age,^48^ gender (male, female, or other),^49^ psychiatric history, duration since infection,^50^ vaccination for COVID‐19,^51^ and time of participation in peer‐support group sessions were collected as covariates. These items were obtained during the participant agreement.

### Intervention

Online peer support consisted of eight sessions, 1 h per session per week. All sessions were conducted using Zoom (Zoom Video Communications, Inc.). At the time of enrollment, each group consisted of three to six participants and two facilitators (M.M. and M.H.): a psychiatric nurse and a clinical psychotherapist, who were supervised by a peer‐support specialist at every session. The details of these sessions are listed in Table 1. The talk topics of each session were decided by the participants and facilitators in session 1 and before each session based on the pooled desired talk topics raised by the participants in the pre‐intervention questionnaire. Examples of talk topics were as follows: how to deal with PASC, psychological distress regarding PASC, difficulty in doing work and household duties, no way to know when PASC will heal, change of life from before to after PASC, and self‐image of PASC. The ground rules of the groups were shared with participants before session 1 to promote safe and warm communication. These ground rules included refraining from disclosing group discussions to individuals outside the group without permission, avoiding the promotion of specific treatments or folk remedies, and refraining from discussions related to politics, ideology, and religion. Participants could attend sessions in ways and lengths that facilitated their attendance, considering the PASC, such as leaving the session, late attendance, and lying down.

**Table 1:** Details of the online peer support sessions.

Session	Topics
1	Announcement of the aim and the experience process of the peer‐support group and the ground rules by facilitators Self‐introduction Determination of talk topics for sessions 2 to 7 Free discussion State impressions of the session one by one Announcement of the next session by facilitators
2–7	Announcement of the aim and process of the peer‐support group and the ground rules by facilitators State recent conditions one by one Discussion regarding the topic and free discussion State impressions on the session one by one Announcement of the next session by facilitators
8	Announcement of the aim and process of the peer‐support group and the ground rules by facilitators State recent conditions one by one Discussion regarding the thoughts on participating in the past sessions State impression on the session one by one Announcement of asking to respond to the post‐intervention questionnaires by facilitators

### Analyses

After Cohen's *d* for estimating the effect size and the Shapiro–Wilk tests for confirming normality were calculated, the pre–post changes in the outcomes were confirmed via paired *t*‐tests. Sensitivity analyses were also performed using the linear mixed model, which confirmed the change after adjusting for loss to follow‐up and covariates, including age, gender, psychiatric history, duration after infection, vaccination for COVID‐19, and the time of participation. Intention‐to‐treat (ITT) and per‐protocol analyses were conducted. Participants who were absent from the session more than four times were identified as dropouts. Significant outcomes were further analyzed with paired‐*t* tests and Cohen's *d* as supplementary analyses, restricted to those without psychiatric history to determine if psychiatric history influenced the changes. Post hoc power analyses for paired‐*t* tests were also performed.

The data of participants who were lost to follow‐up were excluded using paired *t*‐tests and included in the linear mixed method. Quantitative analyses were performed using Stata 18.0. Statistical significance was set at *P* < 0.05.

A thematic analysis approach was employed for qualitative data, focusing on interactions while participating in peer‐support groups and thoughts on participating in peer‐support groups.^52^ First, two authors (M.H. and M.M.) independently coded the anonymized transcripts generated from the statement records after repeatedly reading them. Second, they reviewed their coding and refined it based on their discussions, as needed, to address discrepancies. Third, the codes were sorted into potential themes and subthemes through an iterative process, with both authors (M.H. and M.M.) collaboratively examining the relevance and relationships between the codes. After several weeks, the draft themes, subthemes, and relevant codes were reviewed and refined as required by M.H. and M.M. for precision and reliability. Finally, the other authors reviewed themes, subthemes, and associated transcripts. Microsoft Excel was used for qualitative analyses.

## RESULTS

### Participants

**Figure 1:**
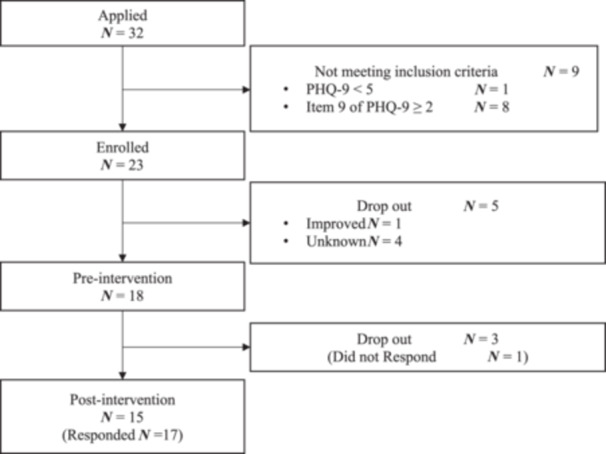
Flow chart.

As shown in Figure 1, 18 of 23 enrolled individuals responded to the pre‐intervention questionnaire, and 15 completed the interventions after three dropped out, although 17 responded to the post‐intervention questionnaire. The attendance numbers for each session in peer‐support groups are detailed in Appendix S1. Although we aimed for at least three participants per group, the actual sessions were performed with two or more participants because some individuals did not attend any sessions. Group C was discontinued due to dropout request. These individuals were not included in the analyses as they did not complete the pre‐questionnaire.

Table 2 presents participant characteristics. The mean age was 41.61 (standard deviation [SD] = 9.50), and 33.3% of the patients were male. The average number of sessions attended was 5.94 (SD = 2.36).

**Table 2:** Participants characteristics.

	Mean or *n* (*n* = 18)	SD or %
Age	41.61	9.50
Gender (male)	6	33.3%
Psychiatric history	12	66.7%
Duration after infection (months)	12.11	6.73
Vaccination	16	88.9%
Times of attendance	5.94	2.36
1	1	5.6%
2	2	11.1%
3	0	0%
4	1	5.6%
5	3	16.7%
6	1	5.6%
7	3	16.7%
8 (all sessions)	7	38.9%

Abbreviation: SD, standard deviation.

### Pre‐post intervention change

The Shapiro–Wilk test for depression, anxiety, loneliness, social withdrawal, and self‐esteem showed no significant differences (*W* = 0.97, *P* = 0.36; *W* = 0.97, *P* = 0.53; *W* = 0.96, *P* = 0.22; *W* = 0.96, *P* = 0.22; *W* = 0.96, *P* = 0.17).

Table 3 shows Cohen's *d* and the results of *t*‐tests. Paired *t*‐tests showed a significant change in the severity of depression regardless of ITT and per‐protocol analysis (pre = 13.33 ± 6.15, post = 11.12 ± 4.55, *t* = 2.26, *P* = 0.04, 1 − β = 0.56; pre = 14.6 ± 5.96, post = 11.73 ± 4.5, *t* = 2.29, *P* = 0.04, 1 − β = 0.57). Cohen's *d* was −0.53 and −0.45 at per‐protocol analysis and ITT analysis. Significant changes in anxiety, loneliness, social withdrawal, and self‐esteem were not observed regardless of ITT or per‐protocol analyses. A similar change in the severity of depression was observed with a larger effect size, although not significant under the condition of limiting the six participants without psychiatric history (pre = 14.17 ± 2.40, post = 10.17 ± 1.58, *t* = 1.83, *P* = 0.06, Cohen's *d* = −0.78, 1 − β = 0.32).

**Table 3:** Pre‐post intervention change.

	Pre		Post		Cohen's *d*	Paired *t*‐test		1 − ß
	Mean	SD	Mean	SD		*t*	*P*	
Intention‐to‐treat analyses								
Depression	13.33	6.15	11.12	4.55	−0.45	2.26	0.04	0.56
Anxiety	8.72	5.48	7.94	4.32	−0.19	1.03	0.16	0.16
Loneliness	6.11	1.78	6.29	2.08	0.03	−0.16	0.88	0.05
Social withdrawal	9.28	5.74	9.64	5.74	0.03	−0.34	0.74	0.06
Self‐esteem	27.00	4.92	25.65	3.97	−0.25	1.19	0.25	0.20
Per‐protocol analyses								
Depression	14.6	5.96	11.73	4.5	−0.53	2.29	0.04	0.57
Anxiety	9.73	5.32	8.6	4.01	−0.23	1.03	0.32	0.16
Loneliness	6.33	1.59	6.33	2.09	0	0.00	1.00	–
Social withdrawal	9.07	6.06	9.4	5.85	0.06	−0.61	0.55	0.09
Self‐esteem	27.07	5.26	25.67	4.13	−0.29	1.26	0.23	0.22

Abbreviation: SD, standard deviation.

Appendix S2 presents the results of sensitivity analyses. A significant change in the severity of depression was observed in crude or adjusted ITT analyses (coefficient = −2.42, standard error [SE] = 1.12, 95% confidence interval [CI] = −4.60–−0.23, *P* = 0.03, Akaike's information criterion [AIC] = 210.77, Bayesian information criterion [BIC] = 216.99; coefficient = −2.47, SE = 1.11, 95% CI = −4.66–−0.28, *P* = 0.03, AIC = 205.27, BIC = 220.82). Similar results were obtained from per‐protocol analyses (coefficient = −2.87, SE = 1.25, 95% CI = −5.32–−0.42, *P* = 0.02, AIC = 180.31, BIC = 185.92; coefficient = −2.87, SE = 1.25, 95% CI = −5.32–−0.42, *P* = 0.02, AIC = 164.45, BIC = 178.47). No significant changes in outcomes, other than depression, were observed.

### Qualitative experiences

For interactions while participating in peer‐support groups, five themes with 10 subthemes were extracted (Table 4): (1) interactions conveying the same feelings, such as having the same experiences and opinions; (2) interactions on how to deal with difficulties through proposals for coping strategies, information exchange, and prompting alternative thinking; (3) interactions showing empathy, including encouragement and praise; (4) interactions that enhance the atmosphere of groups, including idle talk and the presentation of differing opinions; (5) interactions that accommodate physical conditions.

**Table 4:** Themes and statement examples regarding interactions while attending peer‐support groups.

Themes	Subthemes	Statement example
Interactions while participating in peer‐support groups		
Interactions that convey having the same feelings	Claims of having the same experiences	“When I think about it carefully, until around this March, for about a year after I got sick, I was in exactly the same condition as you.”
	Supports for others' opinion	“I also truly thought that maintaining my mental state was the most challenging part…”
Interactions for dealing with their difficulties	Proposals of coping strategies	“If you have the environment to rest and adjust your physical condition, based on my experience, I think the risk of your condition worsening is reduced; that's what I thought after listening.”
	Information exchanges	“(In response to a question about the experience with self‐E Epipharyngeal Abrasive Therapy, a self‐care practice) I bought cotton swabs and tried it, but somehow I couldn't properly insert them into my nose, so I stopped.”
	Promoting alternative thinking	“…, so I think that maybe you should really acknowledge your own efforts, your perseverance, and give yourself some credit for that.”
Interactions of showing empathy	Sympathy	“XXX‐san, I think it must be frustrating not knowing the cause…”
	Encouragement	“So, XXX‐san, I don't think you should feel pathetic at all.”
	Praise	“Listening to XXX‐san's story, I thought it was amazing how you've been inventive in dealing with various things…”
Interactions that enhance the atmosphere	Idle talk	“Your icon of the Java sparrow plushie is super cute.”
	Presentation of a differing opinion	”(In response to the opinion that it might be cured in 30 years) …, leading to growing old and dying with a disabled body.”
Interactions adapting to suit health conditions		“It becomes quite tough to look at the screen for a long time, so I often close my eyes.”
Thoughts on participating in the peer‐support groups		
Emotional supports	Feeling relieved through talking	“After I had a chance to speak, I felt a bit lighter.”
	Pleased by the empathy of others	“I'm really grateful that you understand and empathize with me as if it were your own issue.”
	The pleasure of finding out others have the same feelings	“It was really good to have this kind of opportunity, as it's been difficult to meet people who are going through similar experiences.”
Building a sense of bonding	The group's existence as a support	“I was especially looking forward to later sessions, …, naturally, it feels like my mood has lifted a bit in anticipation of this day.”
	Feeling a sense of solidarity	“For 2 months, I've felt a strong sense of camaraderie with XXX‐san and XXX‐san, as if we've been working together in tandem…”
	Being able to speak honestly	“Since I've been lying to everyone around me, it was a relief that I didn't have to tell any lies today.”
	Glad to encourage someone	“Currently, I'm the one receiving a lot of encouragement, but the fact that I can encourage others is something that really makes me happy.”
Change in perspective	Gaining new insights	“I've received a really good perspective, wondering if even now, there might be things I can do aimed at growth, personal growth.”
	Becoming more positive	“At first, I was really down, like I couldn't see the goal, or I didn't know what to do, but now, I feel more like thinking about what I could try doing next or what to experiment with.”
	Viewing oneself objectively	“Through an objective lens, I've come to reaffirm, by listening to everyone's stories, that there indeed are times when I can't move much and that it's a phase where I'm unable to be active.”
Change in behavior or new actions	Becoming a catalyst for taking new actions	“Hearing about how everyone spends their time, I thought about creating moments to step away from social media and try different ways to spend my time.”
	Gaining new information and strategies	“I'm glad to have learned even a little about everyone's current symptoms and how they communicate to others.”
Feelings of inadequate responses during sessions	Struggling with how to approach others	“Because of that, I was a bit unsure about how to join in the conversation or at what point it would be okay to chime in.”
	Wishing for more exchange of information	“I did think it would have been better if we could have set something like this up sooner and had the opportunity to exchange various pieces of information earlier.”
Adverse effects associated with participation	Negative feelings about the group ending	“I'm definitely anxious about the future, feeling as if I'm losing allies around me; that's what I think.”
	Feelings of tension and fatigue during participation	“Actually, I might have been a bit nervous last week too, because I was extremely tired after the meeting ended, and, yes, I ended up feeling quite drained and had to rest for the rest of the afternoon.”

*Note*: XXX‐san represents the names of the participants.

Participants reported experiencing six themes with 16 subthemes after participating in peer‐support groups (Table 4): (1) emotional support, such as feeling relieved through talking, being pleased by empathy from others, and learning that others have the same feelings; (2) building a sense of bonding, such as feeling the group's existence as a support or sense of solidarity, and the pleasure of speaking honestly and encouraging someone; (3) change in perspective, such as gaining new insights, becoming more positive, and viewing oneself objectively; (4) changes in behavior or new actions, such as becoming a catalyst for taking new actions and gaining new information and strategies; (5) feelings of inadequacy during sessions, such as struggling with approaching others and desiring more information exchange; (6) adverse effects associated with participation, including negative feelings about the group ending, tension, and fatigue during attendance.

## DISCUSSION

To the best of our knowledge, this is the first study to examine changes in mental health conditions among individuals with PASC through online peer support conducted at regularly scheduled times with trained facilitators. A significant change in depression severity was observed from pre‐intervention to post‐intervention. Interactions during online peer support were extracted as follows: conveying the same feelings, dealing with difficulties, showing empathy, enhancing the atmosphere, and adapting to suit health conditions. Participants reported impressions of peer‐support groups, including emotional support, a sense of bonding, a change in perspective, a change in behavior, and new actions through participation. However, they also felt inadequate during the sessions and experienced adverse effects associated with participation.

A small to moderate effect on depression severity was observed from pre‐intervention to post‐intervention, indicating that regularly scheduled online peer support with trained facilitators may be mildly effective for individuals with PASC. This result was consistent with the previous studies providing peer‐support communication services via text message and videos at any given time with anyone as optional survices.^33^, ^34^, ^35^ While similar results were obtained, the processes might be different between previous studies and this study. The peer‐support‐like previous studies could provide crisis interventions and a wide range of opinions, meanwhile closed‐regular sessions, such as in this study, can build bonding among participants through repeated regular contact with the same members,^53^, ^54^, ^55^ resulting in improvement.^56^, ^57^, ^58^ Although the possibility of depression improvement being caused by the passage of time cannot be denied, online peer support may be practical for the mental health of patients with PASC.

Qualitative analyses revealed interactions for conveying the same experiences, empathy, proposal of coping strategies and information, enhancing the atmosphere by idle talk, presenting different opinions, and accommodating their conditions, which were indicated to occur during the sessions. Additionally, individuals with PASC reported experiencing emotional support, a sense of bonding, and a change in perspective and behaviors through online peer support, as well as negative impressions, such as feelings of inadequate responses, and adverse effects, such as negative impressions about group ending and fatigue during attendance. Impressions of a sense of bonding and emotional support were consistent with impressions extracted in previous studies.^29^, ^30^ Impressions such as a sense of bonding and emotional support may promote depression improvement based on the study indicating that such impressions reduce mental health problems in different populations.^56^, ^57^, ^58^ Moreover, interactions like conveying the same experiences and empathy may promote impressions like emotional support and to reduce depression, as sharing experiences and showing empathy generally promote bonding and emotional healing.^59^ The impression of changes in perspective and behavior may also have positive effects on depression, considering that cognitive behavior therapy aims to promote changes in perspectives and behaviors that worsen depression to alternative ones. Interactions such as the proposal of coping strategies and information and the presentation of different opinions might contribute to changes in behaviors and perspectives. On the other hand, negative impressions like feelings of inadequate responses and adverse effects, such as negative feelings about group ending and fatigue during attendance, were newly identified in this study to our knowledge. These impressions may interfere with depression improvement through online peer support, as inadequate supportive communication and perceived isolation affect mental health.^56^, ^60^

No significant reductions were observed in anxiety, loneliness, social withdrawal, and self‐esteem. Qualitative results suggest that the end of the peer‐support groups may have contributed to these outcomes, with participants expressing negative feelings such as anxiety and a sense of loss. The end of the group can be seen as a loss of the emotional support and bonding that were obtained through the sessions, which might have played a role in mitigating such psychosocial problems.^61^, ^62^, ^63^ Based on the absence of cutoff point criteria of them, resulting in pre‐mean of anxiety severity being below the cutoff of clinical anxiety definition, a potential floor‐effect might also exist.^64^ Future intervention studies should consider not limiting the number of sessions to better address these problems and setting criteria. Replication studies should also be performed with larger sample sizes, given that the absence of significant differences might be attributable to low statistical power, resulting in a type Ⅱ error.

Furthermore, online peer support may overcome the limitations of medical resources and accessibility for PASC.^28^ Disseminating online peer support for PASC is essential to establish communities that deliver services. Additional care may be implemented based on negative impressions, such as negative feelings toward ending the group sessions, fatigue after attending, and feelings of inadequate response. For example, regular sessions without an ending, sessions with a shorter duration, and consultation services for frustration experienced during the sessions can be proposed.

This study had some limitations. First, we acknowledge that the change in depression observed in this study may have been caused by time elapsed rather than online peer support due to the single‐arm design. However, a similar significant change was observed after adjusting for time elapsed after infection. Additionally, there were sampling biases, such as being limited to individuals with high adherence because of voluntary agreement and having characteristics such as being close to online communities and social media. Representativeness of participants and robustness of results were limited because of the small sample size. Moreover, the accuracy of the presence of PASC was limited, considering that PASC was self‐reported and was not diagnosed by physicians. Participants may have impressions other than what they stated, especially impressions that were difficult to express in the presence of other participants (e.g., negative impressions of others' attitudes). Qualitative analyses were performed with minimal subjective bias; however, complete elimination of such bias is difficult.

In conclusion, this single‐arm pilot study suggests that online peer support with regular sessions and trained facilitators may be effective for treatment of depression among individuals with PASC. To confirm this effect robustly, a two‐arm randomized controlled trial and a study examining the long‐term effects should be conducted in the future.

## AUTHOR CONTRIBUTIONS

Conceptualization: Daisuke Nishi and Megumi Hazumi. Data collection: Megumi Hazumi, Michi Miyake, and Mayumi Kataoka. Formal analysis: Megumi Hazumi and Michi Miyake. Interpretation of data: all. Original draft preparation: Megumi Hazumi. Review and editing: all authors. Supervision: Daisuke Nishi.

## CONFLICT OF INTEREST STATEMENT

Daisuke Nishi reports personal fees outside of the submitted work from Startia, Inc., en‐Power, Inc., MD.net, and Takeda Pharmaceutical Company, Ltd. The remaining authors declares no conflict of interest.

## ETHICAL APPROVAL STATEMENT

This study was approved by the ethics committee of the National Center of Neurology and Psychiatry (A2023‐010).

## PATIENT CONSENT STATEMENT

The study procedure followed the principles of the Declaration of Helsinki, and informed consent was obtained from all patients.

## CLINICAL TRIAL REGISTRATION

This study was registered in the Japan Registry of Clinical Trials (jRCT1030230154).

## Supporting information

Supporting information.

Supporting information.

## Data Availability

Not available.

## References

[1] Thaweethai T , Jolley SE , Karlson EW , Levitan EB , Levy B , McComsey GA , et al. Development of a definition of postacute sequelae of SARS‐CoV‐2 infection. JAMA. 2023;329:1934–1946. 37278994 10.1001/jama.2023.8823 PMC10214179

[2] Nalbandian A , Sehgal K , Gupta A , Madhavan MV , McGroder C , Stevens JS , et al. Post‐acute COVID‐19 syndrome. Nature Med. 2021;27:601–615. 33753937 10.1038/s41591-021-01283-z PMC8893149

[3] Bello‐Chavolla OY , Fermín‐Martínez CA , Ramírez‐García D , Vargas‐Vázquez A , Fernández‐Chirino L , Basile‐Alvarez MR , et al. Prevalence and determinants of post‐acute sequelae after SARS‐CoV‐2 infection (Long COVID) among adults in Mexico during 2022: a retrospective analysis of nationally representative data. Lancet Reg Health Am. 2024;30:100688. 38327277 10.1016/j.lana.2024.100688 PMC10847769

[4] Bowe B , Xie Y , Al‐Aly Z . Postacute sequelae of COVID‐19 at 2 years. Nature Med. 2023;29:2347–2357. 37605079 10.1038/s41591-023-02521-2 PMC10504070

[5] Seighali N , Abdollahi A , Shafiee A , Amini MJ , Teymouri Athar MM , Safari O , et al. The global prevalence of depression, anxiety, and sleep disorder among patients coping with post COVID‐19 syndrome (long COVID): a systematic review and meta‐analysis. BMC Psychiatry. 2024;24:105. 38321404 10.1186/s12888-023-05481-6 PMC10848453

[6] Li Y , Lam LT , Xiao Y , Qiu Z , Zhang Y . The association between Long‐COVID symptomology, perceived symptom burden and mental health in COVID‐19 patients in Shijiazhuang, China: a population‐based health survey. Front Psychiatry. 2024;15:1332066. 38343619 10.3389/fpsyt.2024.1332066 PMC10853326

[7] Hazumi M , Okazaki E , Usuda K , Kataoka M , Nishi D . Relationship between attitudes toward COVID‐19 infection, depression and anxiety: a cross‐sectional survey in Japan. 2022;22:798. 10.1186/s12888-022-04474-1 PMC9761043 36536342

[8] Kataoka M , Hazumi M , Usuda K , Okazaki E , Nishi D . Association of preexisting psychiatric disorders with post‐COVID‐19 prevalence: a cross‐sectional study. Sci Rep. 2023;13:346. 36611076 10.1038/s41598-023-27405-w PMC9823253

[9] Wang S , Quan L , Chavarro JE , Slopen N , Kubzansky LD , Koenen KC , et al. Associations of depression, anxiety, worry, perceived stress, and loneliness prior to infection with risk of post‐COVID‐19 conditions. JAMA Psychiatry. 2022;79:1081. 36069885 10.1001/jamapsychiatry.2022.2640 PMC9453634

[10] Sirotiak Z , Thomas EBK , Brellenthin AG . Stress, anxiety, and depression severity among individuals with no history, previous history, or current history of long COVID. J Psychosom Res. 2023;175:111519. 37832276 10.1016/j.jpsychores.2023.111519

[11] Kerksieck P , Ballouz T , Haile SR , Schumacher C , Lacy J , Domenghino A , et al. Post COVID‐19 condition, work ability and occupational changes in a population‐based cohort. Lancet Reg Health Eur. 2023;31:100671. 37366496 10.1016/j.lanepe.2023.100671 PMC10287546

[12] Halouani N , Gdoura D , Bouattour O , Turki M , Moussa N , Ellouze S , et al. The social stigma and psychological impact in post COVID 19. Eur Psychiatry. 2023;66:S786–S787.

[13] Scholz U , Bierbauer W , Lüscher J . Social stigma, mental health, stress, and health‐related quality of life in people with long COVID. Int J Environ Res Public Health. 2023;20:3927. 36900938 10.3390/ijerph20053927 PMC10001775

[14] Hazumi M , Kataoka M , Narita Z , Usuda K , Nishi D. Psychological distress after COVID‐19 recovery and subsequent prolonged post‐acute sequelae of COVID‐19: a longitudinal study with 1‐year follow‐up in Japan. *medRxiv* 2024. 10.1101/2024.03.21.24304702 40651324

[15] Righi E , Mirandola M , Mazzaferri F , Dossi G , Razzaboni E , Zaffagnini A , et al. Determinants of persistence of symptoms and impact on physical and mental wellbeing in Long COVID: a prospective cohort study. J Infect. 2022;84:566–572. 35150765 10.1016/j.jinf.2022.02.003 PMC8828388

[16] Shi Y , Strobl R , Apfelbacher C , Bahmer T , Geisler R , Heuschmann P , et al. Persistent symptoms and risk factors predicting prolonged time to symptom‐free after SARS‑CoV‑2 infection: an analysis of the baseline examination of the German COVIDOM/NAPKON‐POP cohort. Infection. 2023;51:1679–1694. 10.1007/s15010-023-02043-6 37231313 PMC10212223

[17] Navas‐Otero A , Calvache‐Mateo A , Calles‐Plata I , Valenza‐Peña G , Hernández‐Hernández S , Ortiz‐Rubio A , et al. A lifestyle adjustments program in long COVID‐19 improves symptomatic severity and quality of life. A randomized control trial. Patient Educ Couns. 2024;122:108180. 38330704 10.1016/j.pec.2024.108180

[18] McGregor G , Sandhu H , Bruce J , Sheehan B , McWilliams D , Yeung J , et al. Clinical effectiveness of an online supervised group physical and mental health rehabilitation programme for adults with post‐covid‐19 condition (REGAIN study): multicentre randomised controlled trial. BMJ. 2024;384:e076506. 38325873 10.1136/bmj-2023-076506 PMC11134408

[19] Philip KEJ , Owles H , McVey S , Pagnuco T , Bruce K , Brunjes H , et al. An online breathing and wellbeing programme (ENO Breathe) for people with persistent symptoms following COVID‐19: a parallel‐group, single‐blind, randomised controlled trial. Lancet Respir Med. 2022;10:851–862. 35489367 10.1016/S2213-2600(22)00125-4 PMC9045747

[20] Jimeno‐Almazán A , Franco‐López F , Buendía‐Romero Á , Martínez‐Cava A , Sánchez‐Agar JA , Sánchez‐Alcaraz Martínez BJ , et al. Rehabilitation for post‐COVID‐19 condition through a supervised exercise intervention: a randomized controlled trial. Scand J Med Sci Sports. 2022;32:1791–1801. 36111386 10.1111/sms.14240 PMC9538729

[21] Jimeno‐Almazán A , Buendía‐Romero Á , Martínez‐Cava A , Franco‐López F , Sánchez‐Alcaraz BJ , Courel‐Ibáñez J , et al. Effects of a concurrent training, respiratory muscle exercise, and self‐management recommendations on recovery from post‐COVID‐19 conditions: the RECOVE trial. J Appl Physiol. 2023;134:95–104. 36476156 10.1152/japplphysiol.00489.2022 PMC9829459

[22] Espinoza‐Bravo C , Arnal‐Gómez A , Martínez‐Arnau FM , Núñez‐Cortés R , Hernández‐Guillén D , Flor‐Rufino C , et al. Effectiveness of functional or aerobic exercise combined with breathing techniques in telerehabilitation for patients with long COVID: a randomized controlled trial. Phys Ther. 2023;103:pzad118. 37658773 10.1093/ptj/pzad118

[23] Hausswirth C , Schmit C , Rougier Y , Coste A . Positive impacts of a four‐week neuro‐meditation program on cognitive function in post‐acute sequelae of COVID‐19 patients: a randomized controlled trial. Int J Environ Res Public Health. 2023;20:1361. 36674117 10.3390/ijerph20021361 PMC9858974

[24] Noda Y , Sato A , Shichi M , Sato A , Fujii K , Iwasa M , et al. Real world research on transcranial magnetic stimulation treatment strategies for neuropsychiatric symptoms with long‐COVID in Japan. Asian J Psychiatr. 2023;81:103438. 36610206 10.1016/j.ajp.2022.103438 PMC9795803

[25] Klírová M , Adamová A , Biačková N , Laskov O , Renková V , Stuchlíková Z , et al. Transcranial direct current stimulation (tDCS) in the treatment of neuropsychiatric symptoms of long COVID. Sci Rep. 2024;14:2193. 38272997 10.1038/s41598-024-52763-4 PMC10810850

[26] National Institute for Health and Care Excellence . COVID‐19 rapid guideline: managing the long‐term effects of COVID‐19. London: National Institute for Health and Care Excellence; 2020. 33555768

[27] Centers for Disease Control and Prevention . Post‐COVID conditions: information for healthcare providers. Atlanta, Georgia: Centers for Disease Control and Prevention. 2024. Available from: https://www.cdc.gov/coronavirus/2019-ncov/hcp/clinical-care/post-covid-conditions.html

[28] Ladds E , Rushforth A , Wieringa S , Taylor S , Rayner C , Husain L , et al. Persistent symptoms after Covid‐19: qualitative study of 114 “long Covid” patients and draft quality principles for services. BMC Health Serv Res. 2020;20:1144. 33342437 10.1186/s12913-020-06001-y PMC7750006

[29] Day HLS . Exploring online peer support groups for adults experiencing long COVID in the United Kingdom: qualitative interview study. J Med Internet Res. 2022;24:e37674. 35468083 10.2196/37674 PMC9128729

[30] Hughes R , Fleming P , Henshall L . Peer support groups after acquired brain injury: a systematic review. Brain Inj. 2020;34:847–856. 32421382 10.1080/02699052.2020.1762002

[31] Suresh R , Alam A , Karkossa Z . Using peer support to strengthen mental health during the COVID‐19 pandemic: a review. Front Psychiatry. 2021;12:714181. 34322045 10.3389/fpsyt.2021.714181 PMC8310946

[32] Ibrahim N , Thompson D , Nixdorf R , Kalha J , Mpango R , Moran G , et al. A systematic review of influences on implementation of peer support work for adults with mental health problems. Soc Psychiatry Psychiatr Epidemiol. 2020;55:285–293. 31177310 10.1007/s00127-019-01739-1

[33] Kataoka M , Hazumi M , Usuda K , Miyake M , Nishi D . Effect of online peer support on mental health among patients of post‐acute sequelae of SARS‐CoV‐2 infection: a systematic review. Research Square. 2024. 10.21203/rs.3.rs-4200253/v1

[34] Vaes AW , Goërtz YMJ , Van Herck M , Machado FVC , Meys R , Delbressine JM , et al. Recovery from COVID‐19: a sprint or marathon? 6‐month follow‐up data from online long COVID‐19 support group members. ERJ Open Res. 2021;7:00141‐2021. 34041295 10.1183/23120541.00141-2021 PMC8012818

[35] Wright H , Turner A , Ennis S , Percy C , Loftus G , Clyne W , et al. Digital peer‐supported self‐management intervention codesigned by people with long COVID: mixed methods proof‐of‐concept study. JMIR Form Res. 2022;6:e41410. 36166651 10.2196/41410 PMC9578526

[36] Lewis M , Bromley K , Sutton CJ , McCray G , Myers HL , Lancaster GA . Determining sample size for progression criteria for pragmatic pilot RCTs: the hypothesis test strikes back! Pilot Feasibility Stud. 2021;7:40. 33536076 10.1186/s40814-021-00770-x PMC7856754

[37] Kroenke K , Spitzer RL , Williams JBW . The PHQ‐9: validity of a brief depression severity measure. J Gen Intern Med. 2001;16:606–613. 11556941 10.1046/j.1525-1497.2001.016009606.x PMC1495268

[38] Muramatsu K , Miyaoka H , Kamijima K , Muramatsu Y , Tanaka Y , Hosaka M , et al. Performance of the Japanese version of the patient health questionnaire‐9 (J‐PHQ‐9) for depression in primary care. Gen Hosp Psychistry. 2018;52:64–69. 10.1016/j.genhosppsych.2018.03.007 29698880

[39] Spitzer RL , Kroenke K , Williams JBW , Löwe B . A brief measure for assessing generalized anxiety disorder. Arch Intern Med. 2006;166:1092–1097. 16717171 10.1001/archinte.166.10.1092

[40] Muramatsu K . Validation and utility of a Japanese version of the GAD‐7. Jpn J Psychosom Med. 2009;51:79.

[41] Igarashi T . Development of the Japanese version of the three‐item loneliness scale. BMC Psychol. 2019;7:20. 30953545 10.1186/s40359-019-0285-0 PMC6449911

[42] Hughes ME , Waite LJ , Hawkley LC , Cacioppo JT . A short scale for measuring loneliness in large surveys. Res Aging. 2004;26:655–672. 18504506 10.1177/0164027504268574 PMC2394670

[43] Lubben JE . Assessing social networks among elderly populations. Fam Community Health. 1988;11:42–52.

[44] Lubben J , Blozik E , Gillmann G , Iliffe S , von Renteln Kruse W , Beck JC , et al. Performance of an abbreviated version of the Lubben Social Network Scale among three European community‐dwelling older adult populations. Gerontologist. 2006;46:503–513. 16921004 10.1093/geront/46.4.503

[45] Kurimoto A , Awata S , Ohkubo T , Tsubota‐Utsugi M , Asayama K , Takahashi K , et al. [Reliability and validity of the Japanese version of the abbreviated Lubben Social Network Scale]. Nihon Ronen Igakkai Zasshi. Jpn J Geriatr. 2011;48:149–157. 10.3143/geriatrics.48.149 21778631

[46] Mimura C , Griffiths P . A Japanese version of the Rosenberg Self‐Esteem Scale: translation and equivalence assessment. J Psychosom Res. 2007;62:589–594. 17467414 10.1016/j.jpsychores.2006.11.004

[47] Rosenberg M . Society and the adolescent self‐image. Princeton, NJ: Princeton University Press; 1965.

[48] Hejazian SS , Sadr AV , Shahjouei S , Vemuri A , Abedi V , Zand R . Prevalence and determinants of long‐term post‐COVID conditions in the United States: 2022 behavioral risk factor surveillance system. Am J Med. 2024;S0002‐9343(24)00090‐1. 10.1016/j.amjmed.2024.02.010 38387538

[49] Colizzi M , Comacchio C , De Martino M , Peghin M , Bontempo G , Chiappinotto S , et al. COVID‐19‐induced neuropsychiatric symptoms can persist long after acute infection: a 2‐year prospective study of biobehavioral risk factors and psychometric outcomes. Ir J Psychol Med. 2024:1–8. 10.1017/ipm.2023.53 38351842

[50] Bota AV , Bogdan I , Razvan DV , Ilie AC , Tudor R , Indries MF , et al. A three‐year cross‐sectional analysis of depression, anxiety, and quality of life in patients with post‐COVID‐19 syndrome. Int J Gen Med. 2024;17:751–762. 38476627 10.2147/IJGM.S453247 PMC10929241

[51] Demko ZO , Yu T , Mullapudi SK , Varela Heslin MG , Dorsey CA , Payton CB , et al. Two‐year longitudinal study reveals that long COVID symptoms peak and quality of life nadirs at 6–12 months postinfection. Open Forum Infect Dis. 2024;11:ofae027. 38449921 10.1093/ofid/ofae027 PMC10917418

[52] Braun V , Clarke V . Using thematic analysis in psychology. Qual Res Psycho. 2006;3:77–101.

[53] Reardon ML , Cukrowicz KC , Reeves MD , Joiner TE . Duration and regularity of therapy attendance as predictors of treatment outcome in an adult outpatient population. Psychother Res. 2002;12:273–285.

[54] Melicherova U , Schott T , Brucker M , Hoyer J , Köllner V . Originalbeiträge (Originals). Psychotherapeutic inpatient depression treatment in open versus closed group format: Depressionsbehandlung in offenen versus geschlossenen psychotherapeutischen Gruppen in der stationären Psychotherapie. Z Psychosom Med Psychother. 2024;70:6–23. 37830880 10.13109/zptm.2023.69.oa6

[55] Yalom ID , Leszcz M . The theory and practice of group psychotherapy (Revised). London, England: Basic Books; 2021.

[56] Cornwell EY , Waite LJ . Social disconnectedness, perceived isolation, and health among older adults. J Health Soc Behav. 2009;50:31–48. 19413133 10.1177/002214650905000103 PMC2756979

[57] Green ZA , Faizi F , Jalal R , Zadran Z . Emotional support received moderates academic stress and mental well‐being in a sample of Afghan university students amid COVID‐19. Int J Soc Psychiatry. 2022;68:1748–1755. 34903066 10.1177/00207640211057729

[58] Ozaki K , Motohashi Y , Kaneko Y , Fujita K . Association between psychological distress and a sense of contribution to society in the workplace. BMC Public Health. 2012;12:253. 22463500 10.1186/1471-2458-12-253 PMC3369557

[59] Jong J , Whitehouse H , Kavanagh C , Lane J . Shared negative experiences lead to identity fusion via personal reflection. PLoS One. 2015;10:e0145611. 26699364 10.1371/journal.pone.0145611 PMC4689389

[60] Clausing D , Fowler ME , Harmon C , Tucker A , Outlaw D , Akce M , et al. Association of emotional support with quality of life, mental health, and survival in older adults with gastrointestinal malignancies—results from the CARE registry. Cancer Med. 2023;12:19102–19111. 37644881 10.1002/cam4.6477 PMC10557900

[61] Shin Y . Moderating effects of friendship on the association between withdrawal loneliness and social anxiety. Family Environ Res. 2015;53:667–675.

[62] Wang J , Mann F , Lloyd‐Evans B , Ma R , Johnson S . Associations between loneliness and perceived social support and outcomes of mental health problems: a systematic review. BMC Psychiatry. 2018;18:156. 29843662 10.1186/s12888-018-1736-5 PMC5975705

[63] Stinson DA , Logel C , Zanna MP , Holmes JG , Cameron JJ , Wood JV , et al. The cost of lower self‐esteem: testing a self‐ and social‐bonds model of health. J Pers Soc Psychol. 2008;94:412–428. 18284290 10.1037/0022-3514.94.3.412

[64] Plummer F , Manea L , Trepel D , McMillan D . Screening for anxiety disorders with the GAD‐7 and GAD‐2: a systematic review and diagnostic metaanalysis. Gen Hosp Psychistry. 2016;39:24–31. 10.1016/j.genhosppsych.2015.11.005 26719105

