# Supplementary material for: Online peer support for mental health in individuals with post‐acute sequelae of COVID‐19: A pre‐post pilot study with mixed methods
Source: PCN Rep. 2024 Aug 18;3(3):e238. doi: 10.1002/pcn5.238 (PMC11330590; doi:10.1002/pcn5.238)
Supplement: Supplementary file 1 — Supporting information. [file PCN5-3-e238-s001.docx]

**S1 Appendix. The attendance number for each session in peer support groups**

|  | **Peer support group** (n) | | | | |
| --- | --- | --- | --- | --- | --- |
|  | **A** | **B** | **C** | **D** | **E** |
| **#1** | 3 | 3 | 2 | 4 | 4 |
| **#2** | 2 | 3 | 2 | 4 | 2 |
| **#3** | 2 | 3 | – | 3 | 3 |
| **#4** | 3 | 5 | – | 3 | 3 |
| **#5** | 3 | 5 | – | 4 | 2 |
| **#6** | 3 | 5 | – | 4 | 3 |
| **#7** | 3 | 3 | – | 4 | 1 |
| **#8** | 2 | 4 | – | 4 | 3 |

–, not held
