# Supplementary material for: Online peer support for mental health in individuals with post‐acute sequelae of COVID‐19: A pre‐post pilot study with mixed methods
Source: PCN Rep. 2024 Aug 18;3(3):e238. doi: 10.1002/pcn5.238 (PMC11330590; doi:10.1002/pcn5.238)
Supplement: Supplementary file 2 — Supporting information. [file PCN5-3-e238-s002.docx]

**S2 Appendix. Pre and post-intervention change based on linear mixed methods**

|  | Crude | | | | | |  | Adjusted | | | | | |
| --- | --- | --- | --- | --- | --- | --- | --- | --- | --- | --- | --- | --- | --- |
|  | **Coef** | **SE** | **95% CI** | **p** | **AIC** | **BIC** |  | **Coef** | **SE** | **95% CI** | **p** | **AIC** | **BIC** |
| Intention-to-treat analyses | | | |  |  |  |  |  |  |  |  |  |  |
| Depression | -2.42 | 1.12 | -4.60 – -0.23 | 0.03 | 210.77 | 216.99 |  | -2.47 | 1.11 | -4.66 – -0.28 | 0.03 | 205.27 | 220.82 |
| Anxiety | -9.93 | 0.97 | -2.82 – 0.97 | 0.34 | 203.20 | 209.42 |  | -1.01 | 0.97 | -2.91 – 0.89 | 0.30 | 197.85 | 213.41 |
| Loneliness | 0.10 | 0.37 | -0.62 – 0.82 | 0.79 | 140.59 | 146.81 |  | 0.07 | 0.37 | -0.65 – 0.79 | 0.85 | 150.40 | 165.96 |
| Social Withdrawal | 0.19 | 0.52 | -0.83 – 1.21 | 0.72 | 190.50 | 196.72 |  | 0.18 | 0.52 | -0.84 – 1.21 | 0.73 | 186.98 | 202.54 |
| Self-efficacy | -1.25 | 0.98 | -3.17 – 0.67 | 0.20 | 199.50 | 205.72 |  | -1.20 | 0.98 | -3.13 – 0.73 | 0.22 | 193.41 | 208.96 |
| Per-protocol analyses | | | |  |  |  |  |  |  |  |  |  |  |
| Depression | -2.87 | 1.25 | -5.32 – -0.42 | 0.02 | 180.31 | 185.92 |  | -2.87 | 1.25 | -5.32 – -0.42 | 0.02 | 164.45 | 178.47 |
| Anxiety | -1.13 | 1.10 | -3.30 – 1.03 | 0.30 | 173.75 | 179.36 |  | -1.13 | 1.10 | -3.30 – 1.03 | 0.30 | 164.94 | 178.96 |
| Loneliness | 0.00 | 0.41 | -0.81 – 0.81 | 1.00 | 120.56 | 126.17 |  | 0.00 | 0.41 | -0.81 – 0.81 | 1.00 | 127.80 | 141.81 |
| Social Withdrawal | 0.33 | 0.55 | -0.74 – 1.41 | 0.54 | 163.54 | 169.14 |  | 0.33 | 0.55 | -0.74 – 1.41 | 0.54 | 154.90 | 168.91 |
| Self-efficacy | -1.4 | 1.11 | -3.58 – 0.78 | 0.21 | 174.01 | 179.62 |  | -1.4 | 1.11 | -3.58 – 0.78 | 0.21 | 164.33 | 178.34 |

Coef, Coefficient; SE, Standard Error; 95% CI, 95% confidence interval; AIC, Akaike information criterion; BIC, Bayesian information criterion
